# Association of Lung Inflammatory Cells with Small Airways Function and Exhaled Breath Markers in Smokers – Is There a Specific Role for Mast Cells?

**DOI:** 10.1371/journal.pone.0129426

**Published:** 2015-06-12

**Authors:** Yvonne Nussbaumer-Ochsner, Jan Stolk, Luiz F. Ferraz da Silva, Annemarie van Schadewijk, Ronald C. de Jeu, Frans A. Prins, Thais Mauad, Klaus F. Rabe, Pieter S. Hiemstra

**Affiliations:** 1 Department of Pulmonology, Leiden University Medical Centre (LUMC), Leiden, The Netherlands; 2 Department of Pathology, Sao Paulo University Medical School, Sao Paulo, Brazil; 3 Department of Pathology, Leiden University Medical Centre (LUMC), Leiden, The Netherlands; 4 Center for Pneumology and Thoracic Surgery, Grosshansdorf Hospital, Grosshansdorf, Germany; University of Athens Medical School, GREECE

## Abstract

**Background:**

Smoking is associated with a mixed inflammatory infiltrate in the airways. We evaluated whether airway inflammation in smokers is related to lung function parameters and inflammatory markers in exhaled breath.

**Methods:**

Thirty-seven smokers undergoing lung resection for primary lung cancer were assessed pre-operatively by lung function testing including single-breath-nitrogen washout test (sb-N2-test), measurement of fractional exhaled nitric oxide (FeNO) and pH/8-isoprostane in exhaled breath condensate (EBC). Lung tissue sections containing cancer-free large (LA) and small airways (SA) were stained for inflammatory cells. Mucosal (MC_T_) respectively connective tissue mast cells (MC_TC_) and interleukin-17A (IL-17A) expression by mast cells was analysed using a double-staining protocol.

**Results:**

The median number of neutrophils, macrophages and mast cells infiltrating the lamina propria and adventitia of SA was higher than in LA. Both MCTC and MC_T_ were higher in the lamina propria of SA compared to LA (MC_TC_: 49 vs. 27.4 cells/mm^2^; MC_T_: 162.5 vs. 35.4 cells/mm^2^; P<0.005 for both instances). IL-17A expression was predominantly detected in MC_TC_ of LA. Significant correlations were found for the slope of phase III % pred. of the sb-N2-test (r_s_= -0.39), for the FEV_1_% pred. (r_s_= 0.37) and for FEV_1_/FVC ratio (r_s_=0.38) with MC_T_ in SA (P<0.05 for all instances). 8-isoprostane concentration correlated with the mast cells in the SA (r_s_=0.44), there was no correlation for pH or FeNO with cellular distribution in SA.

**Conclusions:**

Neutrophils, macrophages and mast cells are more prominent in the SA indicating that these cells are involved in the development of small airway dysfunction in smokers. Among these cell types, the best correlation was found for mast cells with lung function parameters and inflammatory markers in exhaled breath. Furthermore, the observed predominant expression of IL-17A in mast cells warrants further investigation to elucidate their role in smoking-induced lung injury, despite the lack of correlation with lung function and exhaled breath parameters.

## Introduction

Tobacco smoke is the most important risk factor for the development of chronic obstructive pulmonary disease (COPD) [1,2]. Smoking induces an inflammatory response along the trachea-bronchial tree, the lung parenchyma and pulmonary vasculature. The cellular pattern is quite heterogeneous and comprises different inflammatory cells including mast cells [3–6]. Compared to non-smokers, smokers have increased numbers of mast cells in the large airways [7]. Mast cells are composed of mucosal (MC_T_) and connective tissue mast cells (MC_TC_) [8]. Smoking and especially the development of COPD seems to induce a shift in mast cell phenotype with an increase in the percentage of MC_TC_ [6].

The IL-17 cytokine superfamily triggers the production of different cytokines resulting in the recruitment of inflammatory cells. There is increasing evidence for elevated IL-17 expression in chronic obstructive lung disorders [9,10]. IL-17A is not only expressed by T-cells but also other inflammatory cells including mast cells [11] and it is up-regulated by cigarette smoke exposure in human lung tissue explants [12] being likely involved in inflammatory cell recruitment in smoking-induced lung injury. From previous studies in smokers we know that cellular distribution of inflammatory cells within the airways is non-uniform with neutrophils and mast cells being more prominent in the small airways compared to the large airways [13]. Inflammation is associated with the development of smoking-related small airway dysfunction reflected by functional impairment as measured by physiologic tests [14]. The single-breath-N2-test (sbN2-test) non-invasively detects the presence of uneven distribution of ventilation and airway closure in the small airways at a time when not significantly changed, and has been found to be associated with small airways pathology scores [15,16]. Other non-invasive techniques identifying airway abnormalities linked to the effects of smoking comprise the analysis of exhaled breath condensate (EBC) and fractional exhaled nitric oxide (FeNO).

So far, a comprehensive histological analysis of cellular infiltration in the large and small airways of smokers and its correlation with exhaled breath markers and functional (small) airway obstruction has not been investigated within the same study and was therefore the aim of the following study. Furthermore, we were interested whether these inflammatory processes are related to certain subtypes of mast cells and their ability to produce IL17.

## Methods

### Patients and tissue collection

Patients scheduled for surgical resection of primary lung cancer and a positive history of smoking (at least 10 pack years) were prospectively enrolled in this cross-sectional study. Ex-smokers were classified as having stopped smoking for at least 6 months before lung resection. Patients who had received chemotherapy prior to surgery or resection due to other diseases than lung cancer were not included. Spirometry, lung volume measurement using helium dilution and carbon monoxide diffusing capacity were performed preoperatively (within one week before surgery) according to current guidelines [17,18]. A diagnosis of COPD was based on a postbronchodilator FEV_1_/FVC ratio < 0.7 and a relevant medical history. The study was approved by the medical ethics committee of the Leiden University Medical Centre (Leiden, The Netherlands, NCT01145300), and written informed consent was obtained.

### Tissue sampling and processing

One or two central airways and at least 2 samples of peripheral lung tissue were collected as distant as possible from the tumor. Samples were coded and placed in 4% buffered formaldehyde, dehydrated and embedded in paraffin. Sequential 4 μm thick sections were generated and from all blocks, a haematoxylin-eosin stained slide was used to check for the absence of tumor tissue, pneumonia or lung fibrosis.

### Immunohistochemistry

**Fig 1:**
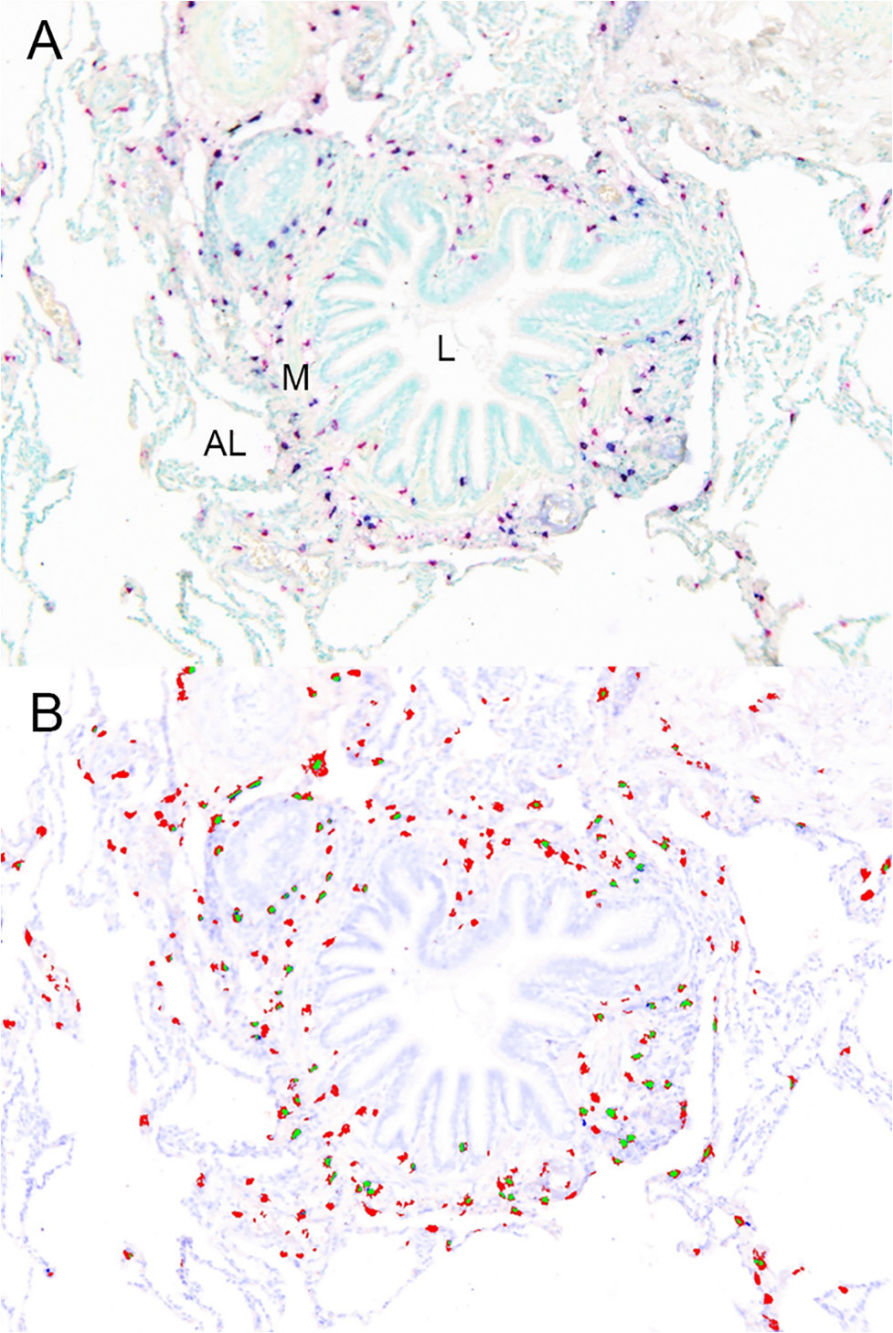
Example of an immunohistochemical staining and its corresponding spectral image. **A** Small airway containing different subtypes of mast cells: MC_T_ (tryptase only) are in pink (Liquid Permanent Red, DAKO), MC_TC_ are double positive for pink and blue (Vector blue, Vector Labs). Section is counterstained with methylene green. AL, alveolar lumen; L, airway lumen; M, smooth muscle. **B** Co-localization using spectral imaging technique: red dots representing MC_T_, green dots representing MC_TC_. Original magnification: x10.

Immunohistochemistry was performed on consecutive tissue sections and slides were stained for T-lymphocytes (CD3, CD4, CD8), B-lymphocytes (CD20), macrophages (CD68), neutrophils (neutrophil elastase; NE) and eosinophils (eosinophil cationic protein; EG2) using specific antibodies (Table 1). Mast cells can be distinguished in mucosal mast cells (MC_T_) that stain only positive for the protease tryptase, and connective tissue mast cells (MC_TC_) that express both tryptase and chymase [8]. For the simultaneous visualization of both MC_T_ and MC_TC_ a double-staining protocol was developed. After deparaffinization, rehydration and heat-induced antigen retrieval in Tris-ethylenediaminetetraacetic acid (pH 9, 97°C, DAKO, Glostrup, Denmark) slides were treated with UltraV Block (Immunologic, Duiven, Netherlands) to block non-specific binding sites. Subsequently, the sections were double-stained using an immuno-alkaline phosphatase method with anti-mouse immunoglobulin conjugated to alkaline phosphatase as a second step. Tryptase-containing mast cells were detected with an anti-tryptase antibody (Table 2) and the chromogen Permanent Red (DAKO, Carpinteria, USA). Next, the remaining MC_TC_ subclass was visualized with an anti-chymase antibody (Table 2). Before the incubation of this antibody, antigen retrieval and treatment with Ultra V Block was repeated for 10 respectively 15 minutes. Colour was developed using the Alkaline Phosphatase substrate kit III (Vector Blue, Vector Laboratories Inc, Burlingame, USA), providing a purple reaction product in double stained cells. For the double-staining with IL-17A, the same pretreatment was used as described above. Slides were incubated with polyclonal goat anti-human IL-17A (Table 2) and detection was performed using biotinylated polyclonal rabbit anti-goat immunoglobulin (DAKO, Glostrup, Denmark), avidin-biotin-peroxidase complex (Vectastain Elite ABC kit; Vector Laboratories) and Permanent Red. Tryptase- respectively chymase-positive cells were detected by Vector Blue as described above. The slides were counterstained with methylene green (Fig 1A).

**Table 1:** Antibodies used for immunohistochemistry.

Antibody	Species	Dilution	Clone	Origin	Secondary antibody
Anti-CD3	Mouse	1:50		DAKO, Glostrup, Denmark	polyAP-Anti-Mouse IgG
Anti-CD4	Mouse	1:50		DAKO, Glostrup, Denmark	polyAP-Anti-Mouse IgG
Anti-CD8	Mouse	1:1000	C8/144B	DAKO, Glostrup, Denmark	polyAP-Anti-Mouse IgG
Anti-CD20	Mouse	1:100	L26	DAKO, Glostrup, Denmark	polyAP-Anti-Mouse IgG
Anti-CD68	Mouse	1:250	PG-M1	DAKO, Glostrup, Denmark	polyAP-Anti-Mouse IgG
Anti-NE	Mouse	1:200		DAKO, Glostrup, Denmark	polyAP-Anti-Mouse IgG
Anti-EG2	Mouse	1:1000		Pharmacia & Upjohn Diagnostics AB, Sweden	polyAP-Anti-Mouse IgG
Anti-tryptase	Mouse	1:20000	G3	Millipore, Temecula, USA	polyAP-Anti-Mouse IgG
Anti-chymase	Mouse	1:1000	CC1	Leica Microsystems, Newcastle, UK	polyAP-Anti-Mouse IgG
Anti-IL-17A	Goat	1:20		R&D Systems, Abindon, UK	Biotinylated Polyclonal rabbit anti-goat IgG

**Table 2:** Patient characteristics.

Male/Female	28/9
Ex-smokers/current smokers	16/21
Age (years)	63±9
Pack years	33±17
Steroid inhaler (yes/no)	13/24
Oral steroid (yes/no)	5/32
COPD (GOLD stage I:II:III:IV)	19 (0:14:4:1)
FEV_1_ (% pred.)	64±15
FEV_1_/FVC (%)	70±12
T_L_CO ml/min/mmHg (% pred.)	73±18
Slope III (% pred.)	246±128
CV/VC (% pred.)	103±37
CC/TLC (% pred.)	113±19

N = 37. Mean±SD. FEV1 = forced expiratory volume in 1 second; FVC = forced vital capacity. T_L_CO = carbon monoxide transfer factor. Slope III = slope of phase III of the nitrogen washout test; CV = closing volume; VC = vital capacity; CC = closing capacity; TLC = total lung capacity.

### Morphological and morphometric analysis

Depending on the availability, 1 or 2 central airways and 1 to 5 small airways from at least 2 samples of peripheral parenchyma were analysed per patient. Central or large airways (LA) were defined as cartilaginous airways, maximum from the third generation. Small airways (SA) were defined by an internal perimeter < 6 mm, and to avoid measurements in tangentially cut airways airways with a short/long diameter ratio <0.33 were excluded from the study. In LA and SA, the following regions were analyzed separately: the lamina propria or lamina propria (IL, area between the epithelial basement membrane and the smooth muscle), the smooth muscle area and the adventitia or adventitia (OL, area outside smooth muscle to the adjacent cartilaginous area in LA respectively to the connection points of the surrounding parenchyma in SA).

Since tryptase, chymase and IL-17A all have a cytoplasmatic localization, we used spectral imaging for detection of colocalization (i.e. the presence of both Permanent Red and Vector Blue in one cell) (Fig 1) [19]. For all large and small airways, 10 digital images were taken at random using a Leica microscope (Leica DM 4000B, Goettingen, Germany), Multispectral imaging system Nuance FX camera and Nuance 2.10.0 software (Cri, Woburn, MA). After loading the spectral characteristics containing the individual spectra of Permanent Red and Vector Blue, the software was used to unmix the spectra in these images. The thresholds for colocalization were defined by two independent investigators after having visually counted the number of double positive cells. The same threshold and spectral settings were used for analysis in all images of according series. The cellular infiltrate was then counted manually by a researcher blinded to the clinical information using Image J 1.43 (National Institute of Health, USA).

### Small airways function

Small airways function was assessed by the single breath nitrogen test (sbN_2_-test) within one week prior to surgery. To minimize the contribution of smooth-muscle contraction, the sbN_2_-test was performed 15 min after administration of 400 μg of salbutamol per metered-dose inhaler connected to a spacer. The measurement was performed using a dry roll-sealing spirometer (Spiroflow; Morgan; Kent, UK) filled with 100% oxygen and equipped with a N_2_ meter (Morgan) connected to the mouthpiece allowing continuous sampling as described previously [15,20,21]. Each patient performed a full slow inspiratory and expiratory slow vital capacity (VC) manoeuvre at a flow rate of approximately 0.5 L/s which was controlled by a mechanical flow regulator. The expiratory N_2_ concentration (%N_2_) was plotted against the lung volume (between TLC and RV) and the slope of the nitrogen alveolar plateau (slope of phase III) was calculated by drawing the best-fit line through phase III of the expiratory volume concentration curve (%N2/L) by two blinded observer. The first departure from this straight line was considered as indicative for airway closure and allowing to calculate closing volume (CV, phase IV) and closing capacity (CC = RV+CV). The measurements were performed twice and only the one with the higher VC was selected for further analysis. The measurements were only accepted if the IVC and expiratory VC during the sbN_2_-test did not differ > 15% or 0.5 L from each other. All volumes from the sbN_2_-test were corrected for body temperature and pressure, saturated with water vapour and the parameters derived from the sbN_2_-test were expressed as percentage of predicted values.

### EBC collection and analysis

All patients were asked to refrain from eating, drinking and smoking for at least 2 hours before the collection of exhaled breath condensate (EBC). Exhaled breath condensate samples were collected within one week prior to the surgery during 10 min of tidal breathing through a single-use disposable RTube device (Respiratory Research, Inc., USA) wearing a nose clip. The collecting tube was enclosed in a sleeve that was precooled at -20°C in a freezer. Approximately 2 ml of condensate was divided in different portions and immediately stored in sterile plastic tubes at -80°C. A 250 μl aliquot of EBC was used for the pH assay. After defrosting and de-aeration by bubbling argon gas through the sample at a rate of 350 ml/min for at least 20 min, pH analysis was done using a thin and sensitive glass electrode and pH meter (Beckman, USA) [22].

8-isoprostane concentrations in exhaled breath condensate were measured as previously described [21] using a specific immunoassay (EIA) kit (Cayman Chemical Company, USA; lower limit of detection 2.7 pg/ml).

### Exhaled NO

Exhaled NO (Fe_NO_) measurements were performed according to the guidelines of the European Respiratory Society and the American Thoracic Society (ATS). The measurements were performed with the NioxMino (Aerocrine, Sweden) at a standardized exhalation flow rate of 50 ml/s within one week prior to surgery. The exhalation manoeuvres were controlled by an audible feedback. Exhaled NO levels were calculated as the mean of 3 technically adequate reproducible measurements.

### Statistical Methods

Data are summarized as mean (SD) for normally distributed variables and as medians (inter quartile range, IQR) for non-normal data distribution. The cellular density is expressed as number of cells per mm^2^. If two or more airways from one patient were examined, mean cell concentration of all airways from this subject was used for statistical analysis. Because the use of sequential sections for e.g. the mast cell subset analyses may be complicated in case of large differences between adjacent sections, we excluded those sections in which this spread was beyond a set limit. Therefore, the density of the tryptase (total mast cells, MC_tot =_ MC_T_+MC_TC_) respectively chymase positive cells (MC_TC_) retained from the IL-17A double staining was plotted against the cellular density of corresponding cells from the tryptase-chymase double staining using Bland-Altman analysis. Only airways with a cellular density within the 95% confidence interval were included in further analysis. Differences between groups were analyzed using Mann-Whitney U-test and paired observation differences using Wilcoxon-matched paired test. Correlations between variables were expressed using the Spearman correlation coefficient (r_s_). Differences and correlations were considered statistically significant at P<0.05. Univariate and multivariate analyses were performed to assess the associations between cellular infiltration and (1) smoking state (ex/current smoker), (2) use of inhaled corticosteroids (ICS) and (3) FEV1% predicted. Variables which significantly correlated with cellular infiltration in the univariate analysis were included in the multivariate analysis. Results are expressed as regression coefficient beta.

## Results

### Patients

From the 38 smokers enrolled in the study, one had to be excluded because histological analysis of the lung tissue revealed cancer infiltration. Finally, tissue from 37 patients was available for analysis. Patient characteristics are shown in Table 2.

### Morphometric analysis

One or two LA were analysed per patient. In the large airways, the median tissue area of the lamina propria was 0.15 mm^2^ (0.1;0.2) and 0.13 mm^2^ (0.10;0.20) for the adventitia. At least one suitable SA was obtained for each patient, with a median of 3 (2;3) SA per patient. The median internal perimeter of the SA was 1.78 mm (1.34;2.67), the median tissue area of the lamina propria 0.05 mm^2^ (0.03;0.20) and of the adventitia 0.12 mm^2^ (0.07;0.20).

**Fig 2:**
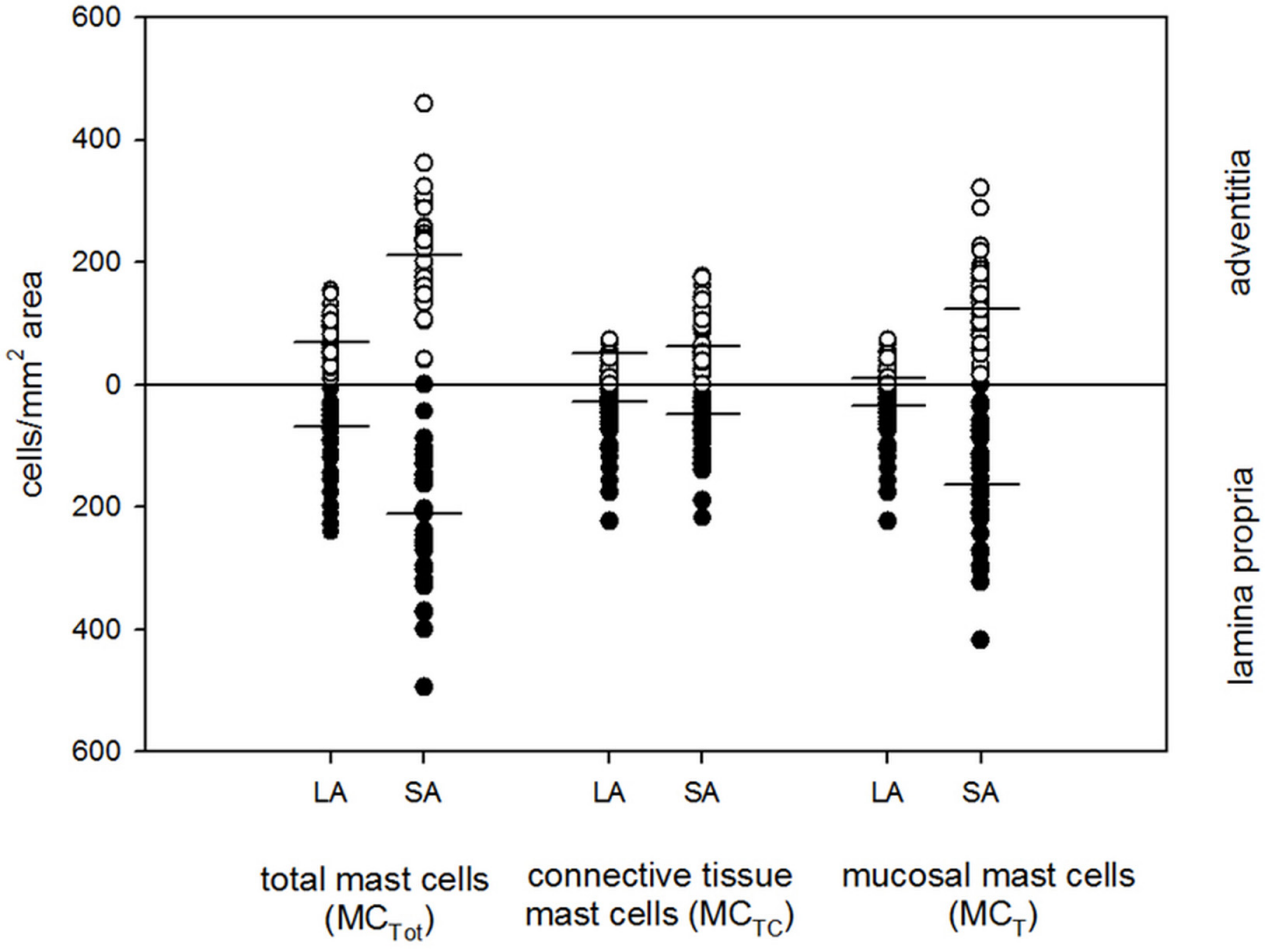
Cellular distribution of mast cells and their subpopulations. Median cellular distribution of mast cells and their subpopulations in the lamina propria and adventitia of large (LA) and small airways (SA) is presented. * P<0.05. The difference between LA and SA in the lamina propria for MC_T_ was 5-fold and 11-fold for the adventitia.

**Fig 3:**
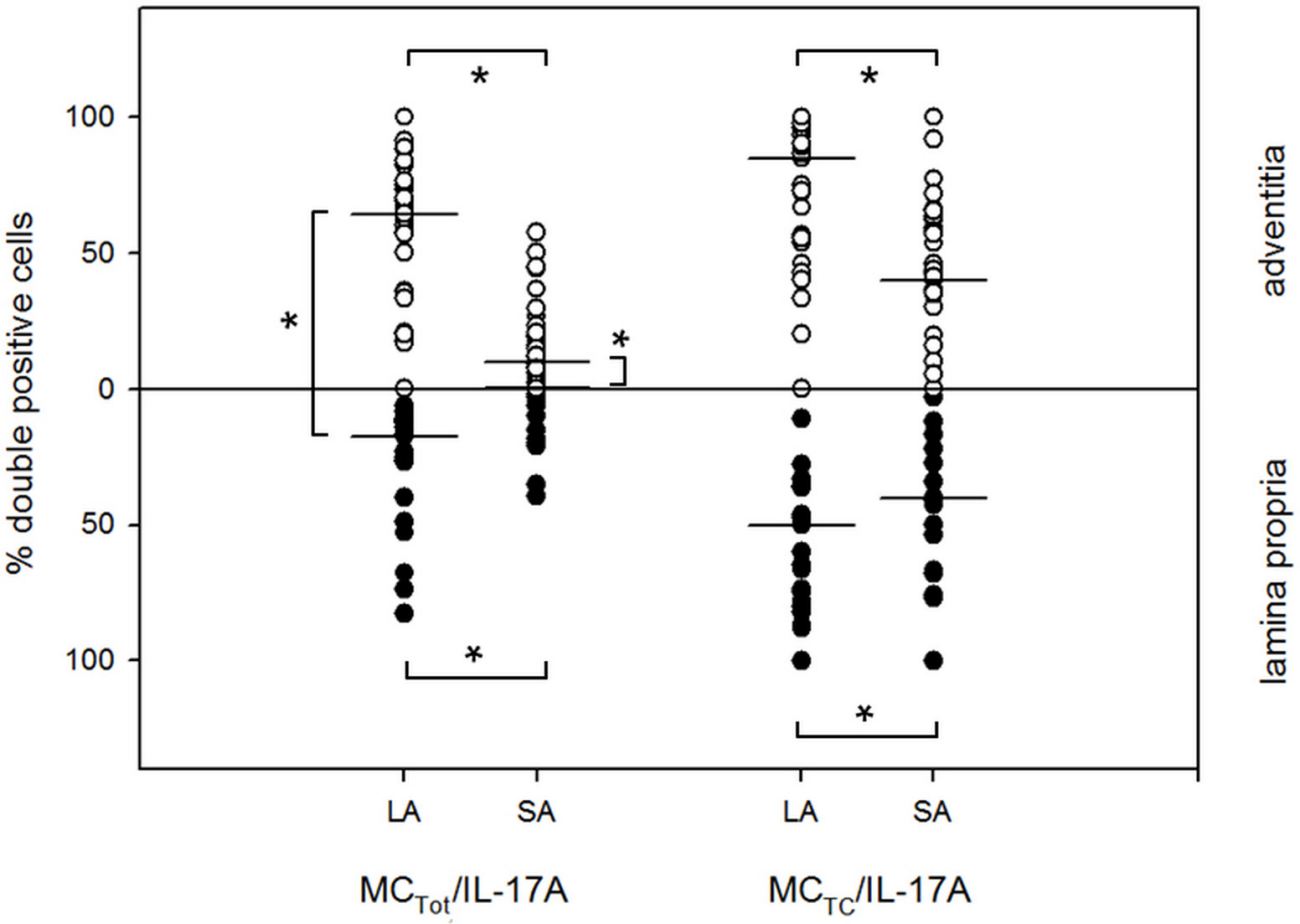
Percentage of IL-17A positive mast cells. Median percentage of IL-17A positive mast cells in the lamina propria and adventitia of large (LA) and small airways (SA) is presented. * P<0.05.

In the whole group, the number of neutrophils, mast cells and macrophages was significantly higher in both the lamina propria and adventitia of SA than in the LA (Table 3). No differences in the cell content infiltrating the LA and SA was found for CD3^+^, CD4^+^, CD8^+^ and CD20^+^ lymphocytes. We next focused on subpopulations of mast cells and observed a significantly higher cell content per square area of both MC_T_ and MC_TC_ in the total wall area (Fig 2). A deeper focus on MC_TC_ cells present in the lamina propria or adventitia showed for the lamina propria 40% (22.9;56.3) in LA and 24.5% (11.2;34.7) in SA; for the adventitia 78.9% (55.3;92) in LA and 39.7% (25.5;50.5) in SA (P<0.05). There was a 5-fold and even higher amount of MC_T_ in the lamina propria respectively the adventitia of SA compared to the LA. Both MC_T_ and MC_TC_ were found to express IL-17A with the percentage of mast cells expressing IL17-A being higher in the LA compared to the SA (Fig 3).

**Table 3:** Cellular distribution of inflammatory cells in smokers.

	Large airways			Small airways		
	lamina propria (cells/mm^2^)	smooth muscle (cells/mm^2^)	adventitia(cells/mm^2^)	lamina propria (cells/mm^2^)	smooth muscle (cells/mm^2^)	adventitia(cells/mm^2^)
Neutrophils (NE)	73.4* # (42.5;126)	0 (0;0)	34.1* (14;62.9)	223.6# (129.7;314.7)	0 (0;0)	116.6 (57.6;260.1)
Macrophages (CD68)	128.4* # (83.5;162)	3.5* (0;29.7)	86.6* (45.2;144.4)	221.3# (141.1;324)	0 (0;0)	406.6 (240;617)
Mast cells (MC_Tot_)	69.1* (53;109.3)	7.6 (0;51)	69.9* (49.1;103.7)	210.4 (148.3;284.2)	0 (0;0)	212.1 (151.6;257.1)
MC_TC_	27.4* # (15;45.7)	0* (0;30.6)	50.6* (25.9;76.5)	49# (24.7;81.9)	0 (0;0)	63.1 (43.6;122.5)
MC_T_	35.4* # (21.3;74.4)	0* (0;8.6)	11* (5.5;28.8)	162.5 (80.6;210.8)	0 (0;0)	122.5 (80.9;163.1)
MC_Tot_/IL-17A	12.2# (0;25.3)	0 (0;16.6)	46.4* (14.2;72.8)	0 (0;24.3)	0 (0;0)	15.7 (5.6;45.7)
% MC_Tot_/IL-17A	17.6* # (0;29.2)	0* (0;20)	64.3* (35.6;75)	0# (0;15.2)	0 (0;0)	9.9 (3.4;20.5)
MC_TC_/IL-17A	19.2# (6.4;38.3)	0* (0;30)	39.9 (16.9;70.4)	12.3# (0;22.2)	0 (0;0)	28.7 (9.1;55.5)
% MC_TC_/IL-17A	50* (33.3;80)	0* (0;80)	84.6* (53.8;100)	40¥ (0;62)	0 (0;0)	40¥ (21;62.3)
CD3^+^ lymphocytes	579.9# (253.8;780.1)	21* (0;72.2)	124.2* (71.2;284.1)	515.7# (441.2;724.9)	0 (0;0)	955.5 (801.7;1164.6)
CD4^+^ lymphocytes	217# (65.3;297.6)	0 (0)	46.1* (2.4;255.1)	280.7# (147.5;332.5)	0 (0;0)	501.5 (386.4;666.7)
CD8^+^ lymphocytes	214.2# (132.6;332.3)	0* (0;45)	73.1* (23.47;237.2)	298.4# (182.9;463)	0 (0;0)	470.8 (339.9;760.3)
CD20^+^ lymphocytes	0# (0;17.4)	0 (0;0)	0* (0;0)	10.6# (0;33.4)	0 (0;0)	49.4 (19;126.8)
Eosinophils (EG2)	21.3# (6.7;58.8)	0 (0;0)	8.4* (0;26.1)	20.4 (0;84.4)	0 (0;0)	24.1 (9;113.4)

N = 37 (non-COPD n = 18, COPD n = 19). Median (IQR).

*P<0.05 vs. corresponding SA within same group.

#P<0.05 vs OL within same group.

¥P<0.05 vs. % MC_Tot_/IL-17A. MC_TC_ = connective tissue mast cells, positive for tryptase and chymase. MC_T_ = mucosal mast cells, only positive for tryptase. MC_Tot_ = all trytpase positive mast cells (MC_TC_ + MC_T_). MC_Tot_/IL-17A = tryptase positive mast cells expressing IL-17A. MC_TC_/IL-17A = chymase positive mast cells expressing IL-17A. % MC_Tot_/IL-17A = percentage of tryptase positive mast cells (MC_TC_ + MC_T_) expressing IL-17A. % MC_TC_/IL-17A = percentage of MC_TC_ expressing IL-17A.

Compared to non-COPD patients, the number of mast cells (MC_Tot)_ and MC_T_ in the lamina propria was significantly lower in COPD patients (MC_Tot_ 261.9 cells/mm^2^ vs. 183 cells/mm^2^ respectively MC_T_ 207.8 vs.101.5 cells/mm^2^). No difference was found for other inflammatory cell types. A significantly higher number of neutrophils in both the lamina propria and adventitia of the small airways was observed in current smokers compared to former smokers (data not shown). In a multiple linear regression analysis no effect of FEV_1_, ICS use or smoking state was seen on mast cell subpopulations or IL17-expression. Only the neutrophilic cellular content of the SA was found to be significantly and independently associated with smoking state (beta coefficient 95.6, CI 12.4 to 178.9, p = 0.03) and FEV1% pred. (beta coefficient -2.7, CI -5.4 to 0.5, p = 0.05).

### Exhaled breath markers / single-breath washout-test

Analysis of EBC revealed a pH of 7.7±0.8 and an 8-isoprostane concentration of 4.8±2.8 pg/ml. Mean exhaled FeNO concentration was 15.7±9.1 ppb. There were no correlations for the EBC pH or FeNO with cellular infiltration of the SA. In the LA, the EBC pH significantly correlated with neutrophils (r_s_ 0.48) and CD20^+^ lymphocytes (r_s_ 0.41) of the adventitia respectively lamina propria, whereas the Fe_NO_ correlated with MC_Tot_ (r_s_ = 0.46) and CD20^+^ lymphocytes (r_s_ = 0.37) in the lamina propria. The 8-isoprostane concentration was significantly correlated with MC_Tot_ cells (r_s_ = 0.44) in the adventitia of small airways (p<0.05). For all other cell types, there were no significant correlations.

In both LA and SA, the content of MC_T_ in the lamina propria correlated significantly with the nitrogen slope % pred. (r_s_ = -0.43 respectively r_s_ = -0.39). There was a significant correlation between MC_T_ in the small airways and both the FEV1% predicted (r_s_ = 0.37) and the FEV_1_/FVC ratio (r_s_ = 0.38, both instances p<0.05). The MC_T_/IL-17A cells and macrophages in LA correlated with the nitrogen slope % pred. (r_s_ = 0.37 respectively r_s_ = 0.49, p<0.05). For both the LA and SA, there were no other correlations for neutrophils and macrophages with FEV_1_% pred., the FEV_1_/FVC ratio, the nitrogen slope or the number of pack years. Significant correlations were found for SA CD4+ lymphocytes and FEV1/FVC ratio (r_s_ = -0.44) respectively FVC % pred (r_s_ = -0.51) and for CD8+ lymphocytes and FVC% pred. in both LA and SA (r_s_ = -0.38 respectively r_s_ = -0.39). In addition, there were no correlations between neutrophils, IL-17A expression and total mast cells.

## Discussion

The present study shows that cellular distribution of inflammatory cells in smokers is heterogeneous with neutrophils, macrophages and mast cells being more abundant in the small airways. In the small airways of smokers, there were more MC_T_ mast cells than MC_TC_ cells, whereas there was no difference in the large airways. Interestingly, this difference was not related to IL-17 expression on mast cells. A further novel finding is the correlation between mast cells and the phase III slope of the sbN2 test pointing towards a role for mast cells in the pathogenesis of airway obstruction. In addition, 8-isoprostane levels in EBC (but not pH) also correlated with mast cell parameters, most notably the MC_T_ subpopulation.

The non-uniform cellular distribution along the tracheo-bronchial tree has been observed by other researchers and is confirmed by our results [13]. The reasons for this observation are speculative; possibly, differences in the local inflammatory milieu might result in a different gradient in cytokines and chemokines. In our study, we specifically focused on the distribution of mast cells subpopulations, which was found to be non-uniform in different compartments of small and large airways in smokers. The number of MC_TC_ in the adventitia of small airways was higher compared to the inner layer (Table 3). The same distribution pattern of MC_TC_ was found by Andersson [6] and Gosman [23] in their smoking control group. In patients suffering from COPD – and especially those with severe stages – the percentage of MC_TC_ in the adventitia of small airways decreased with a shift in relative mast cells densities from the outer to the inner wall layers. Overall, the MC_TC_ population increased in density in all lung compartments with increasing severity of COPD, whereas the MC_T_ population decreased resulting in a net reduction in total mast cell density [6].

Previous studies showed that the number of IL-17A positive cells is increased in the submucosa of central and small airways from COPD patients, indicating an important role of this cytokine in the recruitment of inflammatory cells and even in the development of COPD [10,24]. Our observation that mast cells express a considerable amount IL-17A adds important information to this finding, because a role for mast-cell derived IL-17 has only been proposed in the development of joint destruction in rheumatoid arthritis [25,26]. Why IL-17A expression is mainly related to the MC_TC_ phenotype and is more prominent in the large airways is speculative. Nevertheless, the results of our study suggest that mast cells might be an important player in orchestrating inflammation in the airways by IL-17A-mediated attraction of other inflammatory cells. The observed correlation of the nitrogen slope % pred. with MC_T/_IL-17A density in the LA (r_s_ = 0.37) is in line with this suggestion, although no correlation was found with SA. These findings are underpinned by the fact that IL-17A is upregulated in both non-COPD and COPD lung parenchyma by cigarette smoke exposure using a lung tissue explants culture system [12]. In COPD, increased levels of IL-17A were found in sputum and serum and IL-17A concentration inversely correlated with FEV1% predicted [27]. In a mouse model, smoke exposed IL-17RA^-^/^-^ mice failed to develop emphysema [28] and anti-IL-17 antibodies attenuated airway inflammation in tobacco-smoke exposed mice [29]. These data support the role of IL-17A in smoke-induced airway inflammation and may open a target for new therapeutics.

There is evidence that markers of inflammation are increased in sputum and EBC from smokers [30]. We extended this observation and were interested whether this finding is reflected by impaired small airways function. The sbN2-test is closely related to small airway pathology and has been validated against small airway pathology scores [16]. Small airway narrowing as assessed by the sbN2-test is associated with neutrophilic inflammation in bronchial biopsies and broncho-alveolar lavage in patients with COPD [20]. This could not be confirmed in our study, we only found the correlation for mast cells and the nitrogen slope. This might be attributed to the heterogeneous study population consisting of (ex-) smokers including both COPD and non-COPD patients.

Apart from the correlation between isoprostane concentration and the density of MC_Tot_ respectively MC_TC,_ no further correlation was found for exhaled markers and inflammatory cells. Isoprostanes are produced by reactive oxygen species (ROS)-mediated peroxidation of arachidonic acid by various cell types including mast cells, and are increased in EBC of smokers and COPD patients [31–33]. This correlation further strengthens the special role of mast cells in inducing smoking-induced airway injury.

A potentially confounding aspect in studies performed on surgically resected material from patients with lung cancer is that the tumor itself might affect the local cellular response. However, except for transbronchial biopsies (that are infrequently used only for research purposes) only surgical specimens allow for the examination of peripheral airways. Because we examined tissue at least 2 cm distant from the tumor site we consider it unlikely that the tumor itself biased our results. Unfortunately, no tissue from an adequate non-smoking control group was available arguing that the specific cellular distribution might not be unique to smokers.

The analysis of mast cells was based on two different double staining protocols: one protocol directed at the identification of mast cell subtypes (expression of both tryptase-chymase [MC_TC_] respectively tryptase only [MC_T_]), the other one at the IL-17A analysis. Although we checked the possible error resulting from sequential sections using Bland-Altman analysis (see methods), the density of MC_TC_/IL-17A was higher in both large and small airways than the density of all mast cells expressing IL-17A (MC_T_/IL-17A) (Table 3). Furthermore, the total number of mast cells in the inner layer of small airways as based on the mast cell subtype double staining differed from the total amount of mast cells derived from the IL-17A protocol. This was not seen in the other compartments. A triple staining would certainly have helped to address this problem but was not achieved despite extensive effort. Therefore, no information about tryptase-only positive mast cells expressing IL-17A is available, but based on the results it can be assumed that this is a very small amount. We are aware that other different cell types produce IL-17 and might interact in a more distinct way the inflammatory cascade but in this study, we were interested for the first time whether mast cells are able to express IL-17A in smokers. Because all patients were scheduled for surgical resection and optimal functional control had to be achieved, ICS therapy could not be stopped preoperatively. Even though it is known that ICS decrease cellular inflammation (including mast cells) in the large airways in patients suffering from COPD [34] and IL-17 expression is reduced by dexamethasone in peripheral blood and bronchoalveolar lavage fluid in asthmatics rats [35], no effect of ICS use on different mast cells subpopulations and their IL-17 expression could be seen in our study population using an univariate analysis. The number of patients using systemic corticosteroids was too small to draw a general conclusion on the effect of systemic corticosteroids.

In summary, our results show a differential influx of inflammatory cells in the large and small airways of smokers. They point to a role of IL-17A producing mast cells in smoking-induced lung inflammation that should be further explored, and may reveal a relevant target for directed therapy. Finally, our data show that assessing airway inflammation in smokers only by markers of exhaled breath or small airway testing may be of limited value.
